# Ribosome occupancy profiles are conserved between structurally and evolutionarily related yeast domains

**DOI:** 10.1093/bioinformatics/btab020

**Published:** 2021-01-23

**Authors:** Daniel A Nissley, Anna Carbery, Mark Chonofsky, Charlotte M Deane

**Affiliations:** Department of Statistics, University of Oxford, Oxford OX1 3LB, UK; Department of Statistics, University of Oxford, Oxford OX1 3LB, UK; Department of Statistics, University of Oxford, Oxford OX1 3LB, UK; Department of Statistics, University of Oxford, Oxford OX1 3LB, UK

## Abstract

**Motivation:**

Protein synthesis is a non-equilibrium process, meaning that the speed of translation can influence the ability of proteins to fold and function. Assuming that structurally similar proteins fold by similar pathways, the profile of translation speed along an mRNA should be evolutionarily conserved between related proteins to direct correct folding and downstream function. The only evidence to date for such conservation of translation speed between homologous proteins has used codon rarity as a proxy for translation speed. There are, however, many other factors including mRNA structure and the chemistry of the amino acids in the A- and P-sites of the ribosome that influence the speed of amino acid addition.

**Results:**

Ribosome profiling experiments provide a signal directly proportional to the underlying translation times at the level of individual codons. We compared ribosome occupancy profiles (extracted from five different large-scale yeast ribosome profiling studies) between related protein domains to more directly test if their translation schedule was conserved. Our analysis reveals that the ribosome occupancy profiles of paralogous domains tend to be significantly more similar to one another than to profiles of non-paralogous domains. This trend does not depend on domain length, structural classes, amino acid composition or sequence similarity. Our results indicate that entire ribosome occupancy profiles and not just rare codon locations are conserved between even distantly related domains in yeast, providing support for the hypothesis that translation schedule is conserved between structurally related domains to retain folding pathways and facilitate efficient folding.

**Availability and implementation:**

Python3 code is available on GitHub at https://github.com/DanNissley/Compare-ribosome-occupancy.

**Supplementary information:**

Supplementary data are available at *Bioinformatics* online.

## 1 Introduction

Many protein domains acquire their native structure during synthesis by the ribosome through a process known as co-translational folding (Holtkamp *et al.*, 2015; Nicola et al., 1999; Nissley and O’Brien, 2014; Thommen et al., 2017). Folding during synthesis is intuitively beneficial in that it allows N-terminal sections of proteins to begin acquiring tertiary structure before synthesis of the full-length protein is complete (Frydman *et al.*, 1999). Vectoral folding of this nature helps avoid misfolded conformations (Frydman *et al.*, 1999) and thus leads to more efficient folding of the proteome. Changes to translation speed disrupt protein folding (Cortazzo *et al.*, 2002; Zhang et al., 2009) and function (Noriega et al., 2014; Walter and Johnson, 1994; Zhou et al., 2013) and are thought to be a causal factor in several human diseases including cystic fibrosis (Kim et al., 2015), certain cancers (Sauna and Kimchi-Sarfaty, 2011) and a type of haemophilia (Knobe et al., 2008). Co-translational folding is thus a key part of proteostasis, and its perturbation may lead to the accumulation of misfolded proteins, inducing proteotoxic stress (Nissley and O’Brien, 2016). Given that translation speed influences protein folding and function, it is natural to hypothesize that these speeds are evolutionarily conserved between proteins that share the same fold to aid correct and efficient folding. However, experimentally measuring codon-specific translation speeds was until recently challenging, leading researchers to seek convenient proxies.

The most common proxy for translation speed is rare codon usage. Rare codons tend to have correspondingly rare cognate aminoacyl-tRNA which, by simple chemical kinetic arguments, will increase the dwell time of the ribosome at rare codons relative to commonly occurring codons that are recognized by more common cognate aminoacyl-tRNAs (Fluitt *et al.*, 2007). Many experimental (Buhr *et al.*, 2016; Zhang *et al.*, 2009) and theoretical (Nissley et al., 2016) investigations have found that synonymous codon substitutions can drastically alter the ability of proteins to fold. It makes logical sense, then, that studies of codon usage indicate that clusters of rare codons are conserved in a position-specific fashion (Chaney *et al.*, 2017; Chartier *et al.*, 2012) between homologous protein-coding sequences. One study also found evidence that rare codons are positioned to facilitate co-translational folding, with the odds of finding a rare codon cluster 20–60 codons downstream of a predicted folding intermediate roughly twice the odds of finding a rare codon cluster elsewhere in an mRNA (Jacobs and Shakhnovich, 2017). Rare codons have also been shown to be positioned to facilitate interactions between nascent proteins and the signal recognition particle (Pechmann et al., 2014) as well as with other proteins in the cell (Chartier *et al.*, 2012). However, rare codons are not the only factor that influences translation times: mRNA structure (Hershey *et al.*, 2012), the chemistry of the amino acid being added to the nascent protein (Artieri and Fraser, 2014; Pavlov et al., 2009), mechanical forces generated by nascent proteins (Fritch *et al.*, 2018; Fujiwara *et al.*, 2020; Leininger et al., 2019) and interactions between the nascent protein and the ribosome (Gumbart *et al.*, 2012) are all part of the picture.

Ribosome profiling is a next-generation sequencing technique that produces a signal relative to the number of ribosomes engaged in translation of specific codons across the ensemble of mRNA molecules in cells (Ingolia et al., 2009). The ribosome occupancy at a codon position, assuming experimental biases have been correctly accounted for, should be directly proportional to the mean time required by the ribosome to decode that codon. Ribosome profiling thus provides a more complete proxy for translation speed in living cells than metrics like rare codon usage. One potential downside to ribosome profiling is that no one dataset has sufficient read coverage to provide insight into translation kinetics over the entire translatome. Pooling reads from different experiments to increase read coverage is one way to overcome this shortcoming (Ahmed *et al.*, 2019).

In this article, we find evidence of translation speed conservation based on comparison of ribosome profiling data. Our results demonstrate in a more complete and robust way than all previous studies that translation speed is conserved between structurally and evolutionarily related protein domains.

We compare normalized ribosome occupancy profiles between yeast domains identified by SUPERFAM (Wilson et al., 2009) to be evolutionarily and structurally related. We find that the profiles of these paralogous domains tend to be much more similar to one another than to randomly selected unrelated domains across a Pooled dataset composed of five different ribosome profiling studies (Jan et al., 2014; Nissley *et al.*, 2016; Weinberg et al., 2016; Williams et al., 2014; Young et al., 2015). This trend is also present in the four highest-coverage individual datasets included in our Pooled dataset, with the signal increasing in strength as the number of mapped reads included in our analysis increases. This trend is statistically different from a random control, indicating that biases in the ribosome profiling data alone do not explain our results. Many of the paralogous domains that have highly similar normalized ribosome occupancy profiles also have low DNA sequence identity (<50%), suggesting that translation speed profiles can be conserved over long stretches of evolutionary time.

## 2 Materials and methods

### 2.1 Ribosome profiling datasets included in analysis

The citations and GEO accession numbers of the six individual ribosome profiling datasets from five different studies (Jan *et al.*, 2014; Nissley *et al.*, 2016; Weinberg *et al.*, 2016; Williams *et al.*, 2014; Young *et al.*, 2015) we analysed are provided in Supplementary Table S1. The individual datasets are referred to using the name of the first author of the original study. Results were computed using various different poolings of these six sets of data (Supplementary Table S2). The ‘Pooled’ dataset described below always refers to the dataset that includes reads from all six individual datasets.

### 2.2 Selection of paralogous domain pairs

Reads from each ribosome profiling experiment (Supplementary Table S1) were mapped to the sacCer3 reference transcriptome as described in Nissley *et al.* 2016 and the A-site position within each ribosome-protected fragment determined using an integer-programming method (Ahmed *et al.*, 2019). Only those reads mapped to frame 0 are considered for downstream analysis. The 5,404 domain assignments for yeast strain S288C were cross-referenced with the ribosome occupancy profiles generated from the Pooled dataset and all domains removed that had (i) non-contiguous primary sequence definitions, (ii) less than 100 residues or (iii) less than 70% read coverage.

Pairs of paralogous domains were identified as those domains in the same SUPERFAM family (Wilson *et al.*, 2009). The DNA sequences of each unique pair of related domains were aligned with MUSCLE (Edgar, 2004) and all pairs with less than 30 or greater than 80% DNA sequence identity removed to filter out pairs of distantly and closely related domains, respectively. All pairs of domains passing these criteria were considered for ribosome occupancy profile comparisons, though some are rejected due to the additional criteria described below related to processing raw ribosome profiling read profiles into normalized ribosome occupancy profiles. Ordered locus names, e.g. YEL066W, are used to refer to domains within specific open reading frames in the yeast genome.

### 2.3 Calculation and comparison of ribosome occupancy profiles

The raw ribosome occupancy profiles for pairs of domains were first aligned to the domain pair’s MUSCLE amino acid alignment. Domains with more than ten individual gaps in their alignment or with at least one gap of five positions or more were excluded. Gaps at either end of alignments are not considered in this filtering step. These ‘gappy’ alignments are eliminated to ensure that processed profiles are predominantly composed of experimental data, as gaps in aligned profiles are filled in by univariate spline interpolation on the non-zero positions (see below). The first 40 and last 20 profile positions relative to the full-length gene sequence were then removed to control for biases related to the well-known increase in reads at the 5′ and 3′ ends of the mRNA, respectively (Weinberg *et al.*, 2016). Univariate spline interpolation was used to cover areas with zero read density or at alignment gaps while holding values at all other alignment positions fixed. The resulting profiles were then smoothed with a fifteen-codon moving average (Jacobs and Shakhnovich, 2017; Reuveni et al., 2011) and finally normalized to have an area under the curve of one. Any processed profiles less than 50 positions in length were discarded, leaving 664 pairs of paralogous profiles for the Pooled dataset (Supplementary Table S2).

All pairs of profiles were compared based on their $f_{\mathrm{smf}}$ value. To compute this metric for the similarity between two profiles, the fast, medium and slow positions in each profile are first identified as those positions in the bottom, middle and top thirds of normalized ribosome occupancy. The $f_{\mathrm{smf}}$ value is then computed as the fraction of positions between two normalized profiles with the same classification of fast, medium or slow. Paralogous domain profiles were aligned based on the MUSCLE alignment of their amino acid sequences before calculation of $f_{\mathrm{smf}}$. Visual representations of the processing of raw profiles into normalized ribosome occupancy profiles and the calculation of $f_{\mathrm{smf}}$ are provided in Supplementary Figures S1 and S2, respectively.

### 2.4 Selection of non-paralogous domains for comparisons

Nineteen non-paralogous domains were selected at random for each of the 664 paralogous domain pairs within the Pooled dataset. Non-paralogous read profiles were required to meet the same **≥**70% A-site read coverage criterion as paralogous domains, to be **≥**100 residues in length, and to be within 25 residues of the length of the paralogous domains to which they were compared. Non-paralogous domains were also required to be in a different SCOP class, superfamily and family (Andreeva *et al.*, 2014) from the paralogous domains to which they were compared. Profiles were aligned based on the first common domain position counting from the 5′ end and then truncated at their 3′ end to exactly match the length of the aligned paralogous domain profiles to provide a fair comparison. In cases where the non-paralogous profile was too short, it was rejected and another selected at random. Twenty independent iterations of this selection process were carried out and the paralogous domain profiles ranked against each of these twenty sets of 19 non-paralogous profiles based on $f_{\mathrm{smf}}$ (e.g. Fig. 2a and Supplementary Fig. S2). The results in Figure 2b represent the mean number of paralogous domain pairs in each rank over these twenty random trials.

### 2.5 Calculation of codon usage bias profiles

%MinMax profiles were generated for all domains within the Pooled dataset for which ribosome occupancy profiles were compared. To provide a metric for codon usage bias that has the same single-codon resolution as ribosome profiling data we used a sliding window size of *z* = 1 (Rodriguez et al., 2018). Codon usage frequencies for yeast were downloaded from the CoCoPUTs database (Alexaki *et al.*, 2019). %Min values are reported as negative numbers by convention, so a global additive shift was applied to each profile to set the minimum value within the profile to 1. Following these setup steps, %MinMax profiles were compared in precisely the same fashion as ribosome occupancy profiles.

## 3 Results

### 3.1 Two distantly related paralogous yeast domains have highly similar ribosome occupancy profiles

We constructed the Pooled dataset of ribosome profiling data by combining reads from six ribosome profiling experiments published in five different studies by four different laboratories (Supplementary Table S1) (Jan *et al.*, 2014; Nissley *et al.*, 2016; Weinberg *et al.*, 2016; Williams *et al.*, 2014; Young *et al.*, 2015). Reads were mapped to the sacCer3 reference transcriptome as previously described (Nissley *et al.*, 2016) and the A-site position within each ribosome-protected fragment determined using an integer-programming method (Ahmed *et al.*, 2019). The resulting A-site read counts in the canonical translation frame were then summed across all six experiments. Pairs of structurally related domains within *S. cerevisiae* strain S288C were then identified as those domains within the same SUPERFAM family (Wilson *et al.*, 2009) (i.e. paralogous domains that are structurally and evolutionarily related) and their normalized ribosome occupancy profiles computed as described in Section 2.

**Fig. 1.**
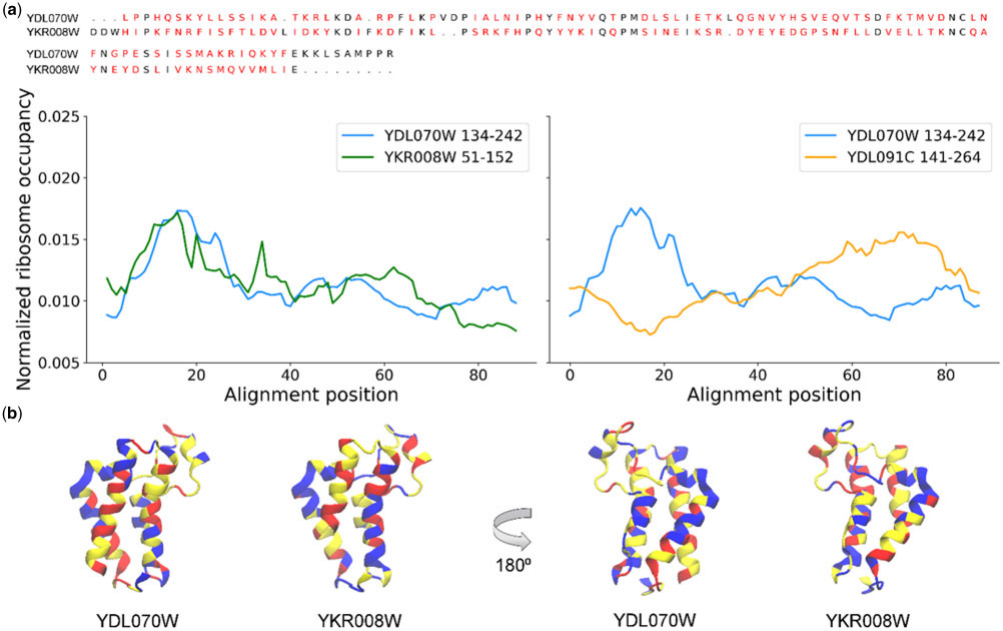
Two yeast Bromodomains have highly similar ribosome occupancy profiles. (**a**) The MUSCLE sequence alignment between the amino acid sequences of the two Bromodomains YDL070W residues 134–242 and YKR008W residues 51–152 (SUPERFAM family 47371, top) has 17% identity. Positions that do not match between both sequences are colored red. The normalized ribosome occupancy profiles for YDL070W residues 134–242 (blue) and YKR008W residues 51–152 (green) were calculated based on the Pooled A-site read dataset as described in Section 2 and plotted as a function of position within the aligned and processed profiles (left panel). The right panel displays the processed ribosome occupancy profiles for YDL070W residues 134–242 (blue) and the randomly selected non-paralogous domain YDL091C residues 141–264 aligned from the first common profile position of their 5′ end (see Section 2). (**b**) PDB ID: 2R0V, which represents YKR008W, colored based on the ribosome occupancy profiles for YDL070W residues 134–242 and YKR008W residues 51–152. Sections of the structures colored red, yellow and blue correspond to the fastest, middle and slowest thirds of translation times within each profile. Note that no trimming, smoothing or normalization of ribosome occupancy profiles was performed in this instance to maintain a length similar to that of the domain itself for the sake of visualization

Figure 1 shows an example of two structurally related domains and their ribosome occupancy profiles. The two Bromodomains YDL070W residues 134–242 and YKR008W residues 51–152 (SUPERFAM family 47371) have highly similar translation speed profiles (Fig. 1a, left panel). The amino acid and DNA sequences of these two domains have just 17% and 44% sequence identity, respectively, indicating a significant amount of evolutionary time has elapsed since the gene duplication event that led to their emergence as paralogous domains. Despite their divergence in both amino acid and DNA sequence, their ribosome occupancy profiles are far more similar to one another than to a randomly selected non-paralogous domain of a similar size (Fig. 1a, right panel). The conservation of ribosome occupancy profiles between these related domains suggests that translation speed may be evolutionarily conserved despite divergence in sequence over evolutionary time.

### 3.2 Ribosome occupancy profiles are conserved between related domains across the yeast translatome

The high degree of similarity between the ribosome occupancy profiles of YDL070W residues 134–242 and YKR008W residues 51–152 raises the question of whether such conservation is a general phenomenon. That is—are ribosome occupancy profiles of related domains more similar to one another than to profiles of unrelated domains, despite divergence in sequence, across the yeast translatome? To answer this question, we generated and compared ribosome occupancy profiles between all pairs of related domains with reasonable read coverage within our Pooled dataset.

**Fig. 2.**
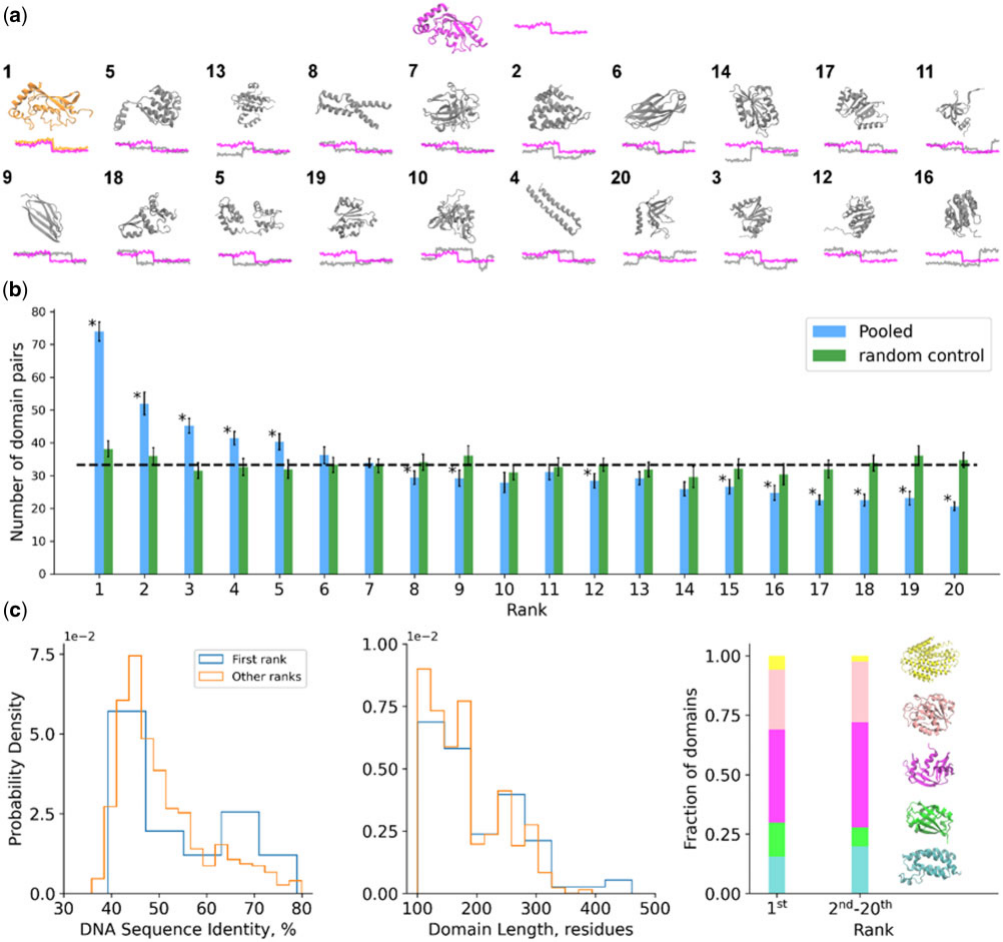
Ribosome occupancy profiles are conserved between related pairs of domains across the yeast translatome. (**a**) Schematic of the comparison procedure for ribosome occupancy profiles of two related domains (magenta and orange). Nineteen unrelated domains of a similar size but in different SUPERFAM superfamilies, families and SCOP classes are also selected (grey structures). The processed ribosome occupancy profiles are then compared on the basis of the $f_{\mathrm{smf}}$ metric. The resulting $f_{\mathrm{smf}}$ scores are then ranked and the position of the pair of related domains in this ranking determined. Numbers represent the position of each domain in the final ranking. In this example, the pair of related domains have the highest $f_{\mathrm{smf}}$ score, indicating they are the most similar out of all pairs of profiles, and they are therefore placed in the first rank. (**b**) The number of paralogous domain pairs from the set of 664 within the Pooled dataset that rank in positions 1^st^ through 20^th^ when compared against one another and against 19 non-paralogous domains as shown in (a) (blue). This analysis was also performed using randomly selected pairs of domains (green). The dotted line indicates the number of paralogous domain pairs expected in each rank if the results are completely random. Error bars are 95% confidence intervals calculated from the results of 20 independent trials. Asterisks indicate ranks for which there is a statistically significant difference between the Pooled results and random control (from permutation test, α=0.05, $1\;x\;10^{6}$ samples). (**c**) (left) Histograms of DNA sequence identity for pairs of paralogous domains in the first rank (blue) and all other ranks (orange) in the first random trial. Subsequent random trials have similar results. (middle) Histograms of domain lengths for pairs of paralogous domains in the first rank (blue) and all other ranks (orange). (right) Stacked barplots indicating the fraction of domain pairs in the first rank and all other ranks that belong to SCOP classes a (α, cyan), b (β, green), c (α+β , magenta), d (α/β, pink), e (multi-domain, yellow), f (membrane, yellow) and g (small proteins, yellow). For simplicity, the rarely occurring classes e, f and g are all colored yellow

Comparisons were performed by first identifying pairs of related domains with sufficient read coverage in their ribosome occupancy profiles. Pairs of domains that are very closely or very distantly related to one another were filtered out by requiring that the DNA sequence identity of domains used in this analysis be between 30% and 80%. Nineteen unrelated domains of similar size were selected for each pair of related domains to serve as an objective comparison set (Fig. 2a). A total of 664 unique pairs of related domains passed all quality control criteria and are included in the analysis (see Section 2). Comparisons between pairs of occupancy profiles were then made by classifying each position in each profile as being in the top, middle or bottom thirds of ribosome occupancy for each individual profile and then computing the fraction of positions in the aligned profiles with the same classification, denoted $f_{\mathrm{smf}}$ (Supplementary Fig. S2). This comparison procedure was carried out twenty times for each paralogous domain pair (once for the paralogous domain pair and nineteen times for the non-paralogous pairs), the results rank ordered and the position of the paralogous pair within the ranking determined. For example, if a paralogous domain pair displays the largest $f_{\mathrm{smf}}$ (most similar profiles) it would be placed in rank 1 as in Figure 2a; if a paralogous domain pair displays the fifth-smallest $f_{\mathrm{smf}}$ (fifth most dissimilar profiles) it is placed in rank 16. This procedure of selecting pairs of related domains along with 19 unrelated domains and comparing their profiles was performed twenty times with different random selections of 19 unrelated domains for each trial. Within the Pooled dataset, 11% of paralogous domain pairs rank in the first position, a 120% increase over the number expected by random chance (Fig. 2b). Only ranks 1, 2, 3, 4 and 5 contain more pairs of paralogous domain pairs than expected by random chance. Qualitatively similar results are obtained for the four highest-coverage individual datasets included in our Pooled dataset (Supplementary Tables S1 and S2, Supplementary Fig. S3). These results indicate that ribosome occupancy profiles are conserved between related yeast domains, suggesting that their translation schedules are conserved.

### 3.3 Accounting for biases in ribosome profiling data

Ribosome profiling experiments suffer from various biases that may cause occupancy profiles to be similar between related domains despite them having dissimilar translation speeds *in vivo*. For example, it is now well-known that the use of chemical agents such as cycloheximide to arrest translation leads to altered occupancy profiles that do not reflect real translation times (Hussmann *et al.*, 2015). Though we have specifically selected ribosome profiling datasets generated without the use of cycloheximide to arrest translation, other biases may be present for which we need to control. To account for such hidden biases, we also performed comparisons between sets of 20 randomly selected domains (random control in Fig. 2b). This selection procedure was performed precisely as for pairs of related domains with two exceptions: (i) selected pairs are not required to be in the same SUPERFAM family, though this may still occur by random chance, and (ii) no DNA sequence identity criterion is applied. A total of 664 random pairs were generated, allowing for fair comparisons. This random control is statistically differentiable from the Pooled dataset results for all ranks except 6, 7, 10, 11, 13 and 14 **(**permutation test, α=0.05, $1\;x\;10^{6}$ samples**)**. The random control trials find a mean of 38.15 pairs of domains in the first rank over twenty trials, somewhat higher than the 33.20 (=664/20) pairs expected if the result was completely random (Fig. 2b). This suggests that while biases and errors are likely present in the ribosome occupancy profiles, these biases alone cannot account for the observed similarity between profiles of related domains in yeast.

### 3.4 Highly similar ribosome occupancy profiles are found regardless of domain size, DNA sequence identity, structural class and amino acid composition

We next investigated the characteristics of paralogous domain pairs with highly similar ribosome occupancy profiles in comparison to those that rank poorly in Figure 2b. Importantly, many pairs of paralogous domains in the top rank have low DNA sequence identity, indicating that their high $f_{\mathrm{smf}}$ values are not primarily due to favorable comparisons between domains with very recent common ancestors (Fig. 2c, left panel). Top-ranked paralogous domain pairs have a similar length distribution to pairs ranked in other positions (Fig. 2c, middle panel), though larger domains are slightly overrepresented in the top rank. There is also no clear dependence on SCOP class, with all four main structural classes (a, b, c and d) found in the top rank and all other ranks in similar proportions (Fig. 2c, right panel). Finally, we compared the amino acid composition of top-ranked domains versus domains with less similar ribosome occupancy profiles. Though overall very similar, top-ranked domain pairs are slightly enriched in His, Trp and Tyr and depleted in Asn (Supplementary Fig. S4). The fact that many top-ranked pairs of domains have low DNA sequence identity indicates that even when sequences have diverged significantly ribosome occupancy profiles remain highly similar. There is no obvious or general difference between pairs of related domains in the top-rank and pairs that rank lower in Figure 2b, suggesting that conservation of translation speed profiles between related domains is a general phenomenon.

## 4 Discussion

Our results indicate that ribosome occupancy profiles produced from ribosome profiling data are conserved between pairs of paralogous domains in yeast. The similarity of these profiles is apparent at the level of individual pairs of related domains (Fig. 1a) and across the set of all paralogous domains in yeast (Fig. 2b) with acceptable read coverage and sequence alignments.

Three hypotheses can explain in part or in whole why ribosome occupancy profiles are conserved between structurally similar domains. First, ribosome occupancy profiles may be conserved due to the influence of translation speed on co-translational folding. Structurally similar domains are likely to fold by similar pathways, meaning that perturbation of the translation speed profile may hinder their folding process and reduce the fitness of the protein. A second hypothesis, which is really a set of hypotheses, is that translation speed is not under selection at all, but factors like mRNA structure that influence translation speed are under selection. Evolutionary pressure on mRNA structure would lead to similar mRNA sequences between paralogous domains and, due to the relationship between codon usage and translation speed, similar ribosome occupancy profiles, despite the fact that the root cause is not related to translation speed. This second hypothesis is not cleanly separable from the co-translational folding hypothesis because mRNA structure, along with many other factors, influences translation speed. A third hypothesis is that we are considering domains that are too closely related, such that the paralogous domain sequences we compare have had too little evolutionary time to diverge and we are effectively comparing profiles to themselves. This third hypothesis is unlikely given the range of sequence identity found for pairs of related domains in the first rank of results (Fig. 2c, left panel). Decoupling the first two hypotheses will provide more insight into the processes underlying conservation of ribosome occupancy profiles between evolutionarily related domains.

Co-translational folding and thus translation speed is often thought to be more important for larger domains with more complex folding landscapes and for domains with more β character, as β-sheets often require forming hydrogen bond networks between portions of domains that are disparate in primary sequence. However, our results indicate no clear difference in domain length, amino acid composition, SCOP structural class or sequence identity between paralogous domains with highly similar profiles and those with dissimilar profiles (Fig. 2c). We have also found that many pairs of paralogous domains rank poorly, and some compare less favorably than all 19 randomly selected unrelated domains (Fig. 2b). This raises a key question: if conservation of ribosome occupancy profiles between related domains is a general phenomenon, why do some pairs of even closely related domains result in poor comparisons*?*

One possible explanation is the high degree of noise inherent to ribosome profiling data and the low coverage found for many coding sequences. Comparing ribosome occupancy profiles generated for the same domain between the Williams and Weinberg ribosome profiling datasets reveals that some domains rank poorly even when compared to themselves between different datasets, though many more domains are found in the first rank than when pairs of related domains are compared within one dataset (Supplementary Fig. S5). This result suggests that ribosome occupancy profiles of individual domains are highly similar between different datasets but not identical. It may be the case that the signal for evolutionary conservation between related domains will become stronger as more high-quality ribosome profiling data becomes available; indeed, as we increase the total number of reads included in our analysis the signal becomes increasingly clear (Supplementary Fig. S6). Increasing sequence similarity also leads to an increasingly strong result (Fig. 2b, Supplementary Figs S5 and S7).

Data with high coverage over the entire translatome will result in more domain pairs with viable read coverage, allowing for extension of our method to more domains with more diverse sequence identity. When more domains are included in the analysis a trend may emerge between protein properties such as SCOP class or domain size and conservation of ribosome occupancy. We also computed codon usage bias profiles using the %MinMax algorithm (Rodriguez *et al.*, 2018) and compared them for the same sets of domains for which ribosome occupancy profiles were compared. We found related domain pairs are most likely to be in ranks 1 or 2 (Supplementary Fig. S8). This result is expected, as previous studies have reported various levels of conservation of codon usage (Chaney *et al.*, 2017; Jacobs and Shakhnovich, 2017). Our results suggest that, at least for the 664 pairs of domains for which we have compared both %MinMax and ribosome occupancy profiles, ribosome occupancy is more strongly conserved between related domains. Our results also indicate that only 1 in 3 pairs of domains with highly conserved %MinMax profiles also have highly conserved ribosome occupancy profile (Supplementary Fig. S8, inset). This suggests that comparison of codon positions alone may provide an incomplete picture of real translation kinetics. Our results show that entire ribosome occupancy profiles are conserved between structurally and evolutionarily related proteins. This result offers strong evidence that translation schedule is important for preserving folding pathways for proteins with similar structures.

One obvious extension of our methods is to compare ribosome occupancy profiles between orthologous proteins in different organisms. Unfortunately, while a general consensus has been reached about how to best process yeast ribosome profiling data, analysis for other organisms remains less clear, and even the best datasets remain low coverage in comparison to the best yeast datasets (Mohammad et al., 2019). We compared ribosome occupancy profiles between our Pooled yeast dataset and *E. coli* ribosome profiling data from Mohammed and co-workers (Mohammad *et al.*, 2019). We found that while some individual pairs of related domains have highly similar profiles (Supplementary Fig. S9) between the two organisms, too few pairs of related domains can be compared to provide confidence that this similarity is differentiable from random chance.

If translation schedule is critical to directing folding along optimal pathways, even evolutionarily unrelated proteins with similar folds (i.e. proteins that have undergone convergent evolution) will have similar translation speed profiles. It may be interesting to test this hypothesis in the future using ribosome profiling data.

A deeper understanding of the relationship between conservation of translation schedule and folding pathways may prove important in several areas of protein science. For example, as the quantity of high-quality ribosome profiling data increases it may be possible to extract characteristic translation schedule fingerprints for individual structural motifs [e.g. Greek key (Hutchinson and Thornton, 1993)]. This more detailed understanding of the relationship between translation schedule and structure could then be used for the rational design of proteins with robust co-translational folding characteristics for efficient folding *in vivo*. Conservation of translation schedule between related proteins also has implications for the recombinant expression of proteins. It is now common practice to harmonize the codon usage of the coding sequence to be expressed to match the codon usage of the expression organism (Angov *et al.*, 2008). Our results suggest that matching the translation schedule to preserve the endogenous co-translational folding pathway may result in an even higher fraction of correctly folded, functional protein.

In summary, our results indicate that ribosome occupancy profiles are conserved between structurally related yeast domains. We hypothesize that ribosome occupancy (and thus translation schedule) is conserved to preserve efficient co-translational folding pathways. As more high-quality ribosome profiling data become available more detailed translation schedule trends may be revealed.

## Supplementary Material

btab020_Supplementary_Data
