# Supplementary material for: Ribosome occupancy profiles are conserved between structurally and evolutionarily related yeast domains
Source: Bioinformatics. 2021 Jan 23;37(13):1853–9. doi: 10.1093/bioinformatics/btab020 (PMC8317121; doi:10.1093/bioinformatics/btab020)
Supplement: btab020_Supplementary_Data [file btab020_supplementary_data.docx]

**Supplementary Information: Ribosome occupancy profiles are conserved between structurally and evolutionarily related yeast domains**

Daniel A. Nissley, Anna Carbery, Mark Chonofsky, Charlotte M. Deane*

Department of Statistics, University of Oxford, Oxford, OX1 3LB, UK

*****to whom correspondence should be addressed

**Table S1. Ribosome profiling data sets used in this study**

| **Data set (labeled by first author name)** | **Year of publication** | **Number of replicates** | **GEO study** | **Accession numbers of samples used** |
| --- | --- | --- | --- | --- |
| Jan (Jan et al. 2014) | 2014 | 1 | GSE61012 | GSM1495525 |
| Williams (Williams et al. 2014) | 2014 | 1 | GSE61011 | GSM1495503 |
| Young (Young et al. 2015) | 2015 | 1 | GSE69414 | GSM1700885 |
| Weinberg (Weinberg et al. 2016) | 2016 | 1 | GSE53268 | GSM1289257 |
| Nissley (Nissley et al. 2016) | 2016 | 2 | GSE75322 | GSM1949550  GSM1949551 |

**Table S2. Number of related domain pairs and their characteristics in data sets analysed**

| **Data set** | **Pairs of related domains** | **Unique families** | **Unique superfamilies** |
| --- | --- | --- | --- |
| Nissley1 | 5 | 5 | 5 |
| Nissley2 | 37 | 17 | 17 |
| Young | 134 | 30 | 29 |
| Jan | 272 | 88 | 75 |
| Williams | 408 | 116 | 95 |
| Weinberg | 420 | 118 | 100 |
| Nissley1 + Nissley2 | 71 | 28 | 26 |
| Nissley1 + Nissley2 + Young | 160 | 65 | 57 |
| Nissley1 + Nissley2 + Young + Jan | 398 | 111 | 93 |
| Nissley1 + Nissley2 + Young + Jan + Williams | 590 | 142 | 118 |
| Pooled (Nissley1 + Nissley2 + Young + Jan + Williams + Weinberg) | 664 | 150 | 124 |


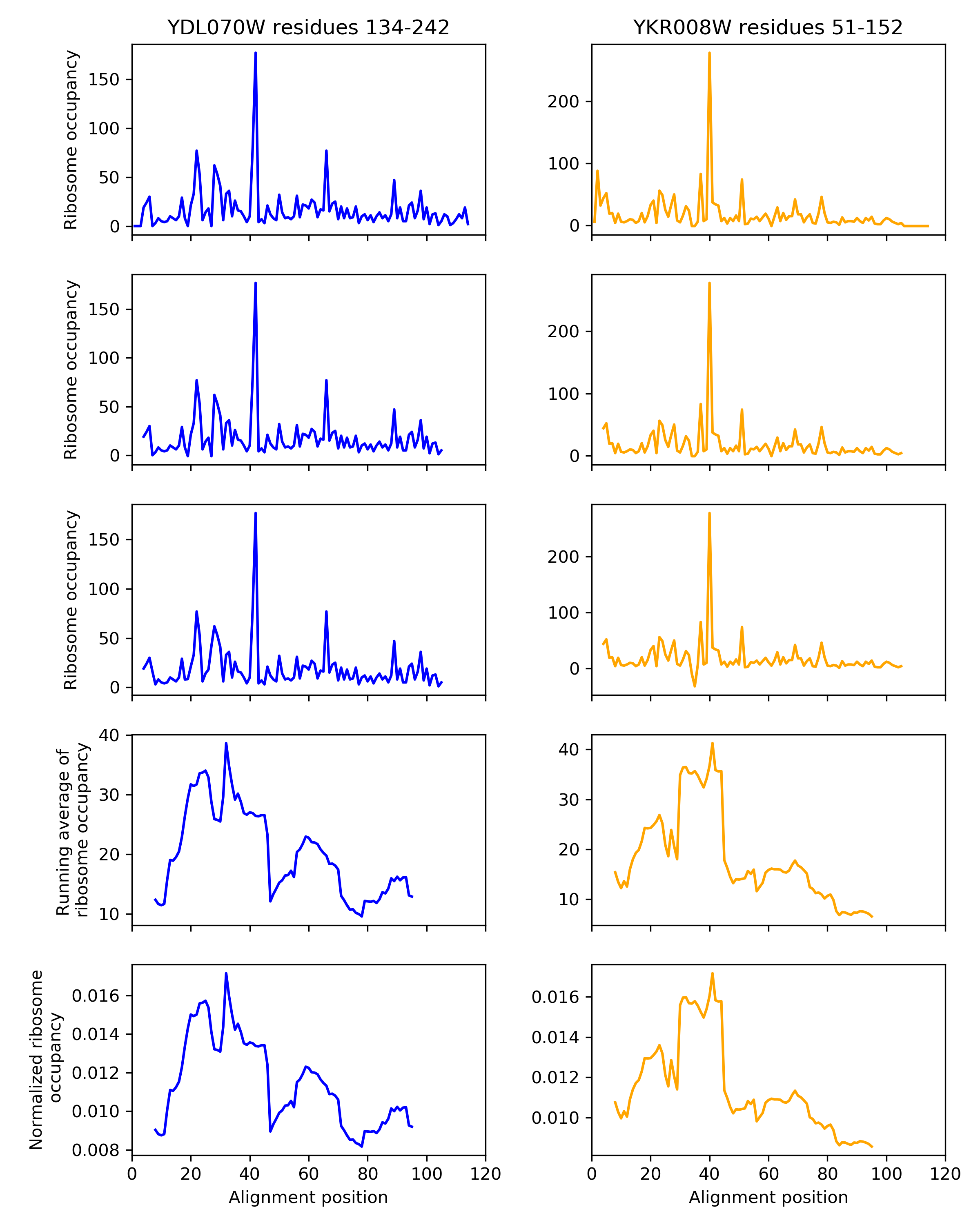


**Figure S1**. **Sample ribosome occupancy profile processing steps for YDL070W residues 134-242 and YKR008W residues 51-152.** The raw ribosome occupancy profiles (first row) are taken as the number of A-site reads in frame 0 at each domain position; positions at gaps in the alignment are set to zero. The profiles are then trimmed (second row) to remove positions that correspond to the first 40 or last 20 codons (see Methods) and also to remove positions at either end with a raw value of 0.0 resulting from missing reads or from gaps in the domain amino acid alignment with MUSCLE. Internal positions with zero reads are then splined (row three) with all other positions with a non-zero read count held constant. The profiles are then smoothed with a 15-position moving average (row four) and then finally normalized (fifth row). These normalized ribosome occupancy profiles are then ready for comparison.


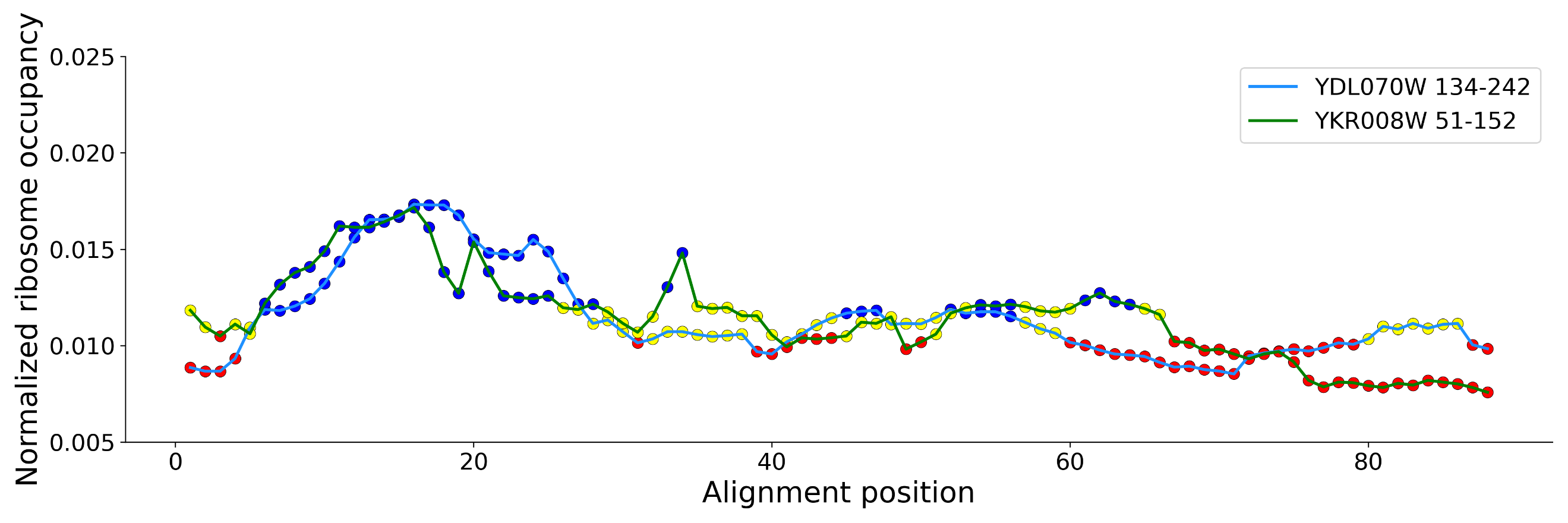


**Figure S2**. **Sample of** $\boldsymbol{f}_{\mathbf{smf}}$ **comparison procedure.** Profiles are compared based on their $f_{\mathrm{smf}}$, which is the fraction of positions in the profiles that receive the same classification of slow, medium, or fast. Points in the above plots that are slow, medium, or fast are colored blue, yellow, and red, respectively. In this instance 52 of 88 alignment positions match, giving $f_{\mathrm{smf}}=0.59$.


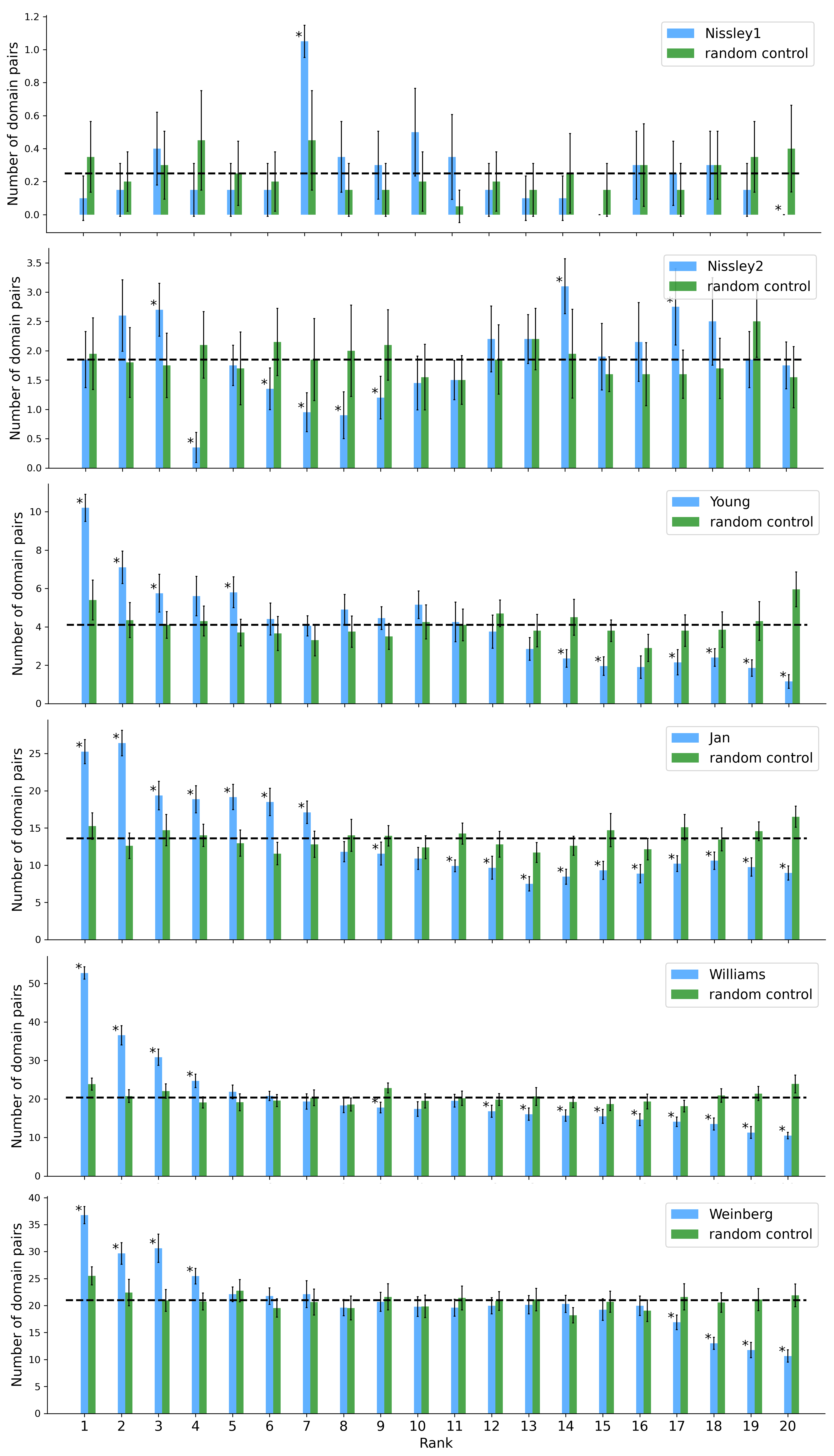


**Figure S3**. **Comparisons between related domain ribosome occupancy profiles for all six individual ribosome profiling data sets**. Nissley1 and Nissley2 refer to the two replicates from Nissley *et al*. 2016 (see Table S1). In each case the random control represents analysis for a total number of random pairs equal to the number of pairs of related domains reported in Table S2. Asterisks indicate ranks for which there is a statistically significant difference between the data set results and the random control (from permutation test, $\alpha=0.05$, $1 x {10}^{6}$ samples).

**
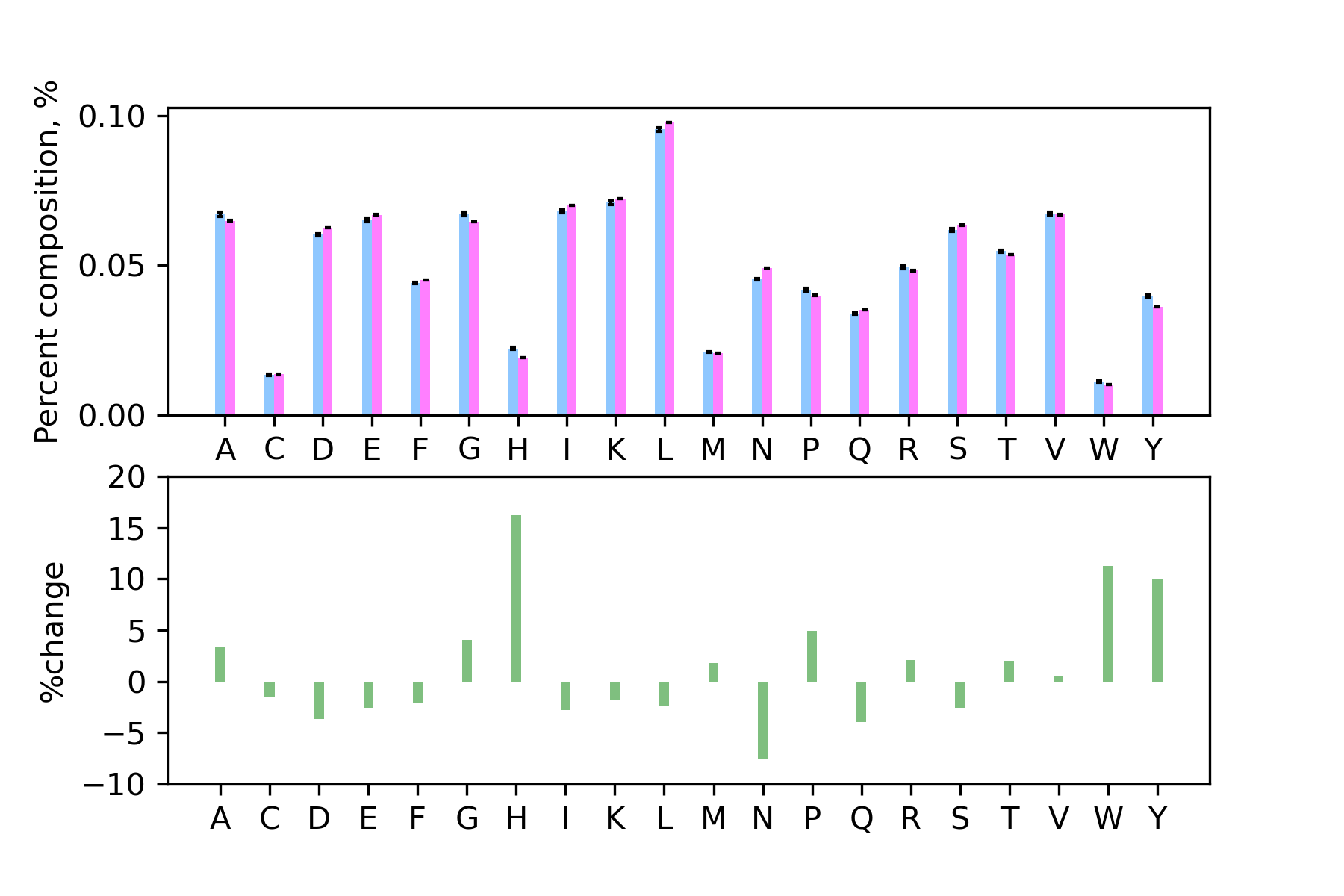
**

**Figure S4**. **Comparison between amino acid composition of domains in the top rank versus domains in all other ranks.** (Top) Percent composition by amino acid of top-ranked domains (blue) and all other domain pairs (magenta). Error bars are 95% confidence intervals computed over each of 20 random trials using the Pooled ribosome profiling data set as input. (Bottom) Percent change between top rank and other rank amino acid compositions.


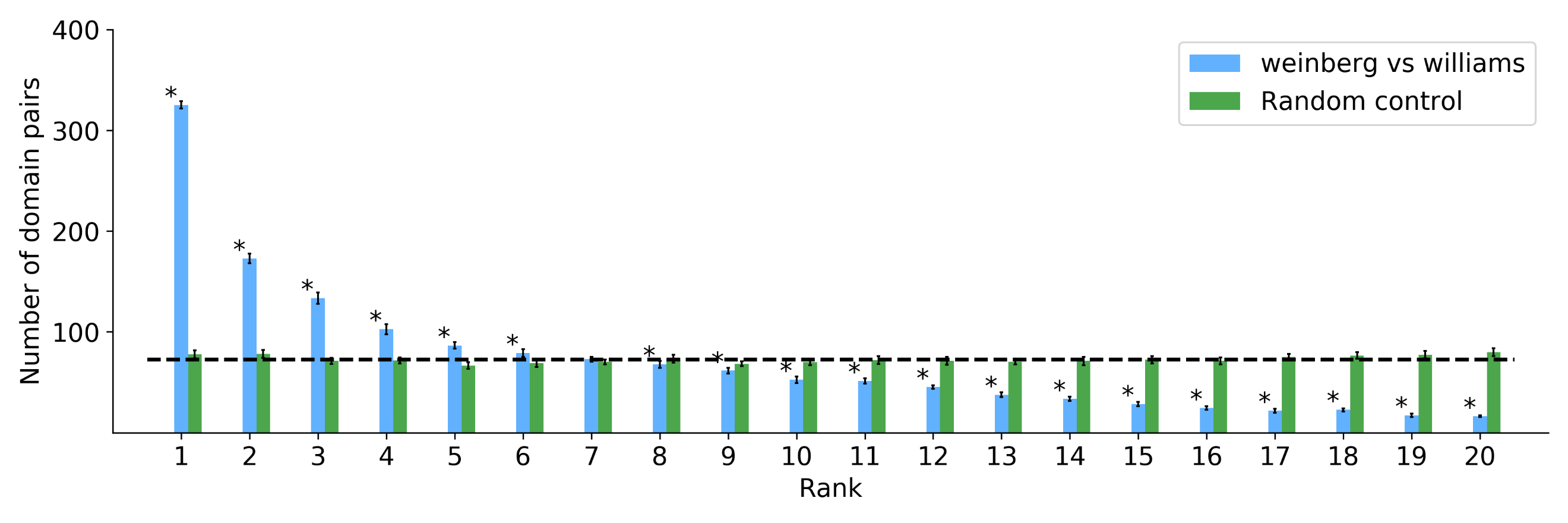


**Figure S5. Comparison between ribosome occupancy profiles for the same domain between Weinberg and Williams data sets.** Comparisons were carried out by comparing the ribosome occupancy profiles for domains within the Williams data set to the profile for the same domain in the Weinberg data set and to 19 randomly selected profiles within the Weinberg data set. Random controls were performed exactly as described for pairs of related domains. A total of 1,427 pairs of ribosome occupancy profiles were compared between the two data sets. Asterisks indicate ranks for which there is a statistically significant difference between the data set results and the random control (from permutation test, $\alpha=0.05$, $1 x {10}^{6}$ samples).


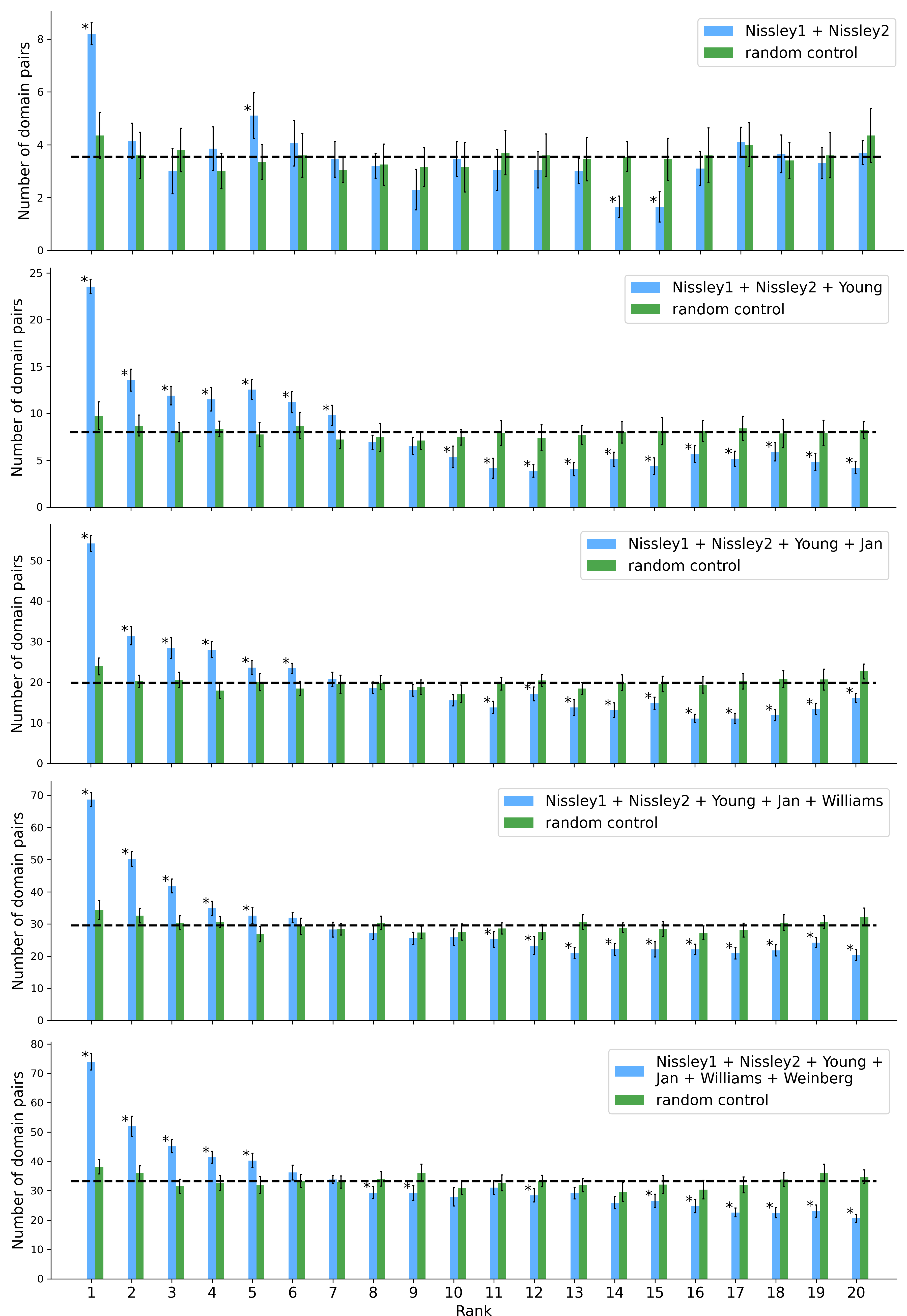


**Figure S6**. **Comparisons between related domain ribosome occupancy profiles for different possible poolings of ribosome profiling data sets**. Nissley1 and Nissley2 refer to the two replicates from Nissley *et al*. 2016 (see Table S1). In each case the random control represents analysis for a total number of random pairs equal to the number of pairs of related domains reported in Table S2. Asterisks indicate ranks for which there is a statistically significant difference between the data set results and the random control (from permutation test, $\alpha=0.05$, $1 x {10}^{6}$ samples).


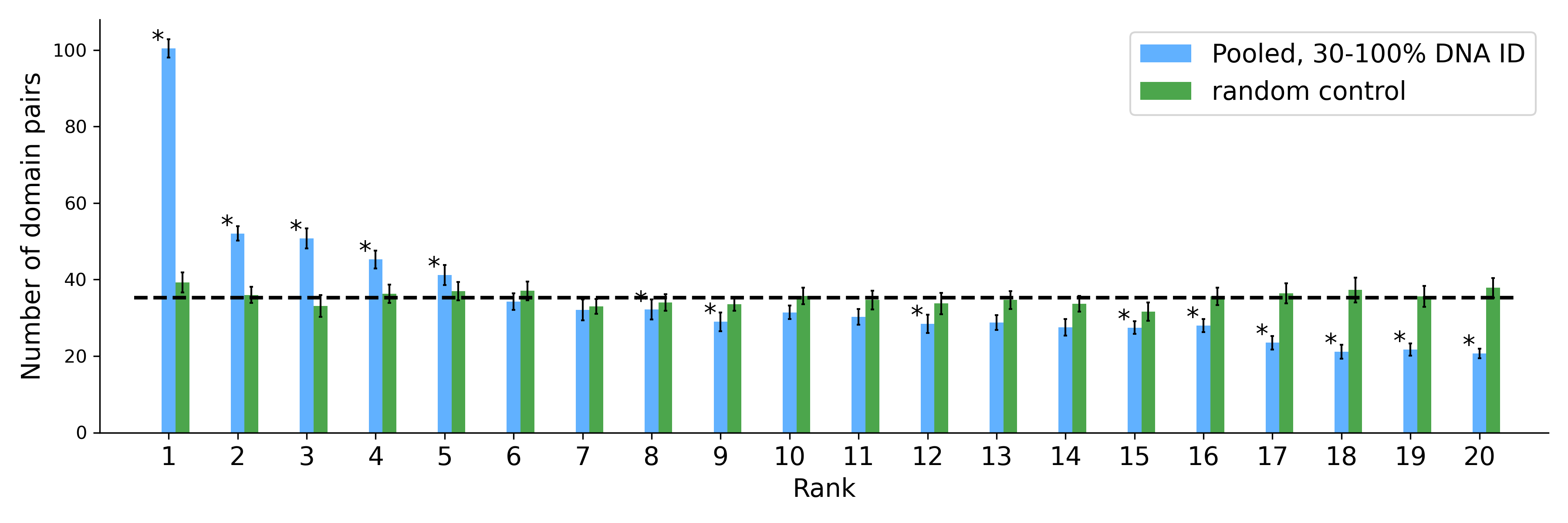


**Figure S7. Comparisons between related domain ribosome occupancy profiles for Pooled data set using all pairs with 30-100% DNA sequence identity**. Asterisks indicate ranks for which there is a statistically significant difference between the data set results and the random control (from permutation test, $\alpha=0.05$, $1 x {10}^{6}$ samples). Calculations are performed exactly as those presented in Figure 2b but with the set of domain pairs with DNA sequence similarity 80-100% included.


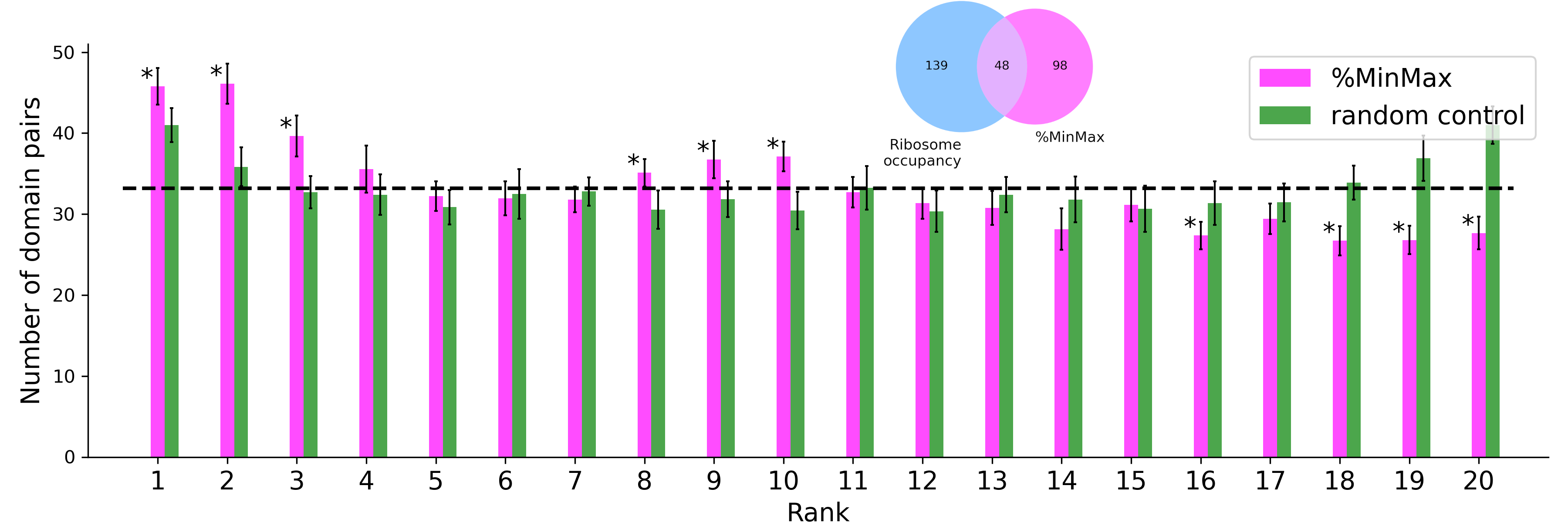


**Figure S8**. Comparisons of %MinMax profiles between pairs of related domains. The same set of 664 domain pairs whose ribosome occupancy profiles are compared in Figure 2b are considered. Asterisks indicate ranks for which there is a statistically significant difference between the data set results and the random control (from permutation test, $\alpha=0.05$, $1 x {10}^{6}$ samples). Subset Venn diagram represents the overlap between the sets of domain pairs whose profiles are placed in the first rank at least once over 20 random trials. Roughly 1 in 3 (48 of 146 pairs) are found in the first rank in both the ribosome occupancy and %MinMax comparisons.


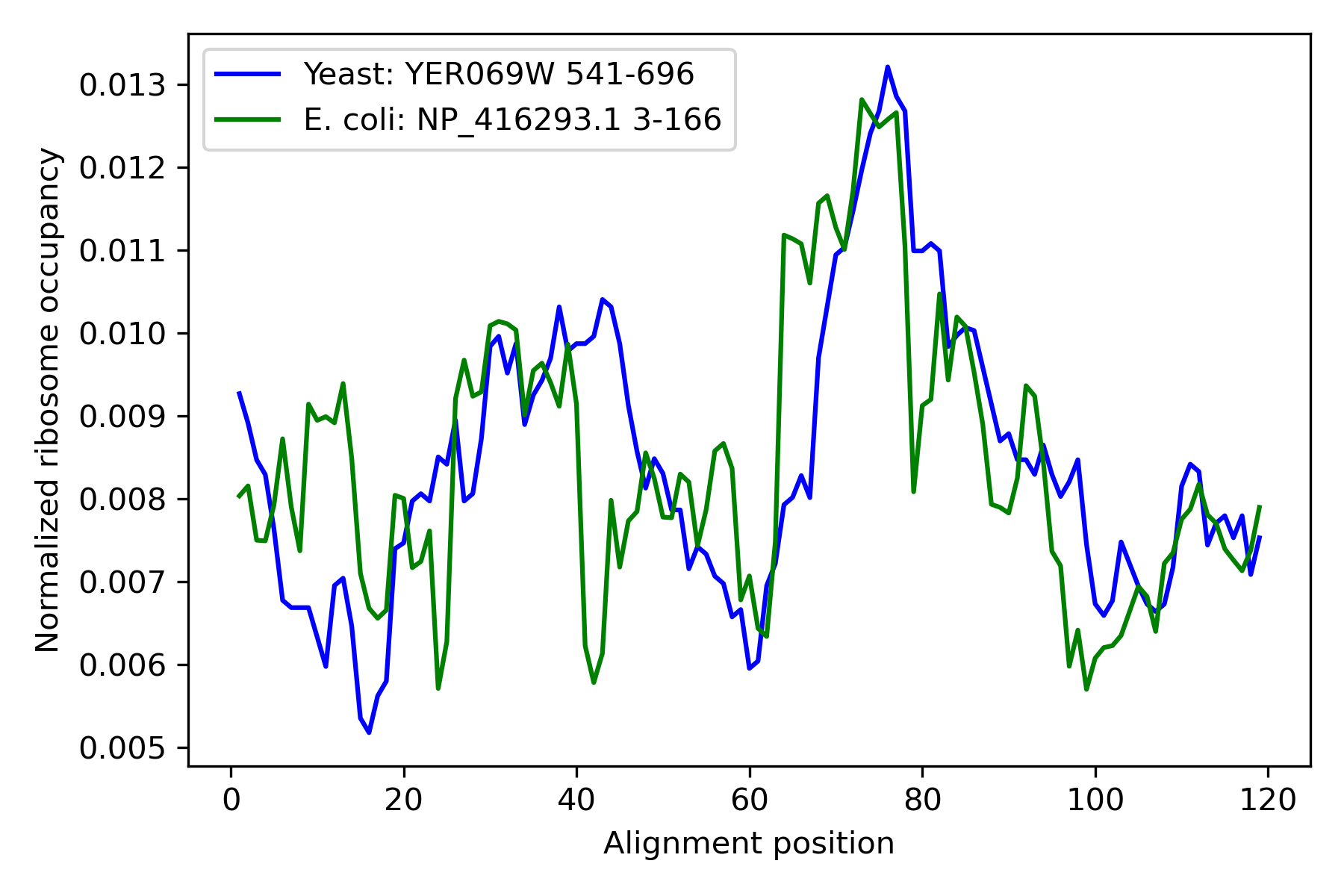


**Figure S9**. Comparison between ribosome occupancy profiles of the two NAD(P)-binding Rossmann-fold domains YER069W 541-696 and NP_416293.1 3-166 from yeast and *E. coli*, respectively. Yeast ribosome occupancy was determined from the Pooled data set (see Tables S1 and S2) while *E. coli* ribosome occupancy were generated from Mohammed *et al.* 2019 data.
